# Risk factors associated with mortality from pneumonia among patients with pneumoconiosis

**DOI:** 10.1186/s40557-016-0103-6

**Published:** 2016-04-07

**Authors:** Bum Seak Jo, Jongin Lee, YounMo Cho, Junsu Byun, Hyoung Ryoul Kim, Jung Wan Koo, Jun Pyo Myong

**Affiliations:** Department of Occupational and Environmental Medicine, College of Medicine, The Catholic University of Korea, Banpo-daero 222, Seoul, Seocho-gu 137-701 Republic of Korea

**Keywords:** Pneumoconiosis, pneumonia, mortality

## Abstract

**Background:**

The aim of this study was to evaluate factors associated with increased risk of mortality from pneumonia among patients with pneumoconiosis.

**Methods:**

Medical records of 103 pneumoconiosis patients hospitalized for pneumonia were investigated. Seven patients who had lung cancer or other malignancy and 13 patients with insufficient medical record were excluded. Two female patients were excluded due to small number to analyze. The subjects were divided into two groups by clinical outcome of pneumonia, the deceased group and the survival group. The two groups were compared in terms of age, smoking history, episode of recent pneumonia, concomitancy of interstitial fibrosis or fungal ball infection, extent of small opacities, grade of large opacities and results of spirometry. Multiple logistic regression was applied to determine the association between these variables and mortality from pneumonia.

**Results:**

The deceased group showed more frequent history of recent pneumonia (*p* = 0.006), higher prevalence of interstitial fibrosis (*p* = 0.007) and longer hospitalization period (*p* = 0.044). The proportion of subjects who had decreased FVC, less than 70 % of predicted value, was higher in the deceased group (*p* < 0.001). In multiple logistic regression, after adjusting age, smoking history, recent pneumonia, fungal ball, large opacity, profusion and FVC (or FEV_1_) less than 70 % of predicted value, history of recent pneumonia, concomitancy of interstitial fibrosis, type of pneumoconiosis and fungal ball presented statistically significant association with mortality from pneumonia.

**Conclusions:**

The concomitancy of fungal ball or interstitial fibrosis, history of recent pneumonia within last 90 days, type of pneumoconiosis, FVC less than 70 % of predicted value, FEV_1_ less than 70 % of predicted value presented statistically significant association with mortality from pneumonia. More attention should be given to patients who have such factors when treating pneumonia with pneumoconiosis.

## Background

Studies on natural history or survival analyses of patients with pneumoconiosis have been constantly carried out during the past several decades [1–8]. There is controversy between several prognostic factors and its outcomes, however, the decreased pulmonary function [1, 2], radiographic findings of pneumoconiosis (such as higher grade of profusions or large opacities in accordance with International Labor Organization (ILO) classification) [3–7], smoking [8] and tuberculosis [4] is well known risk factors for progression to death among patients with pneumoconiosis.

Pneumonia is one of the most common disease entities that requires hospitalization and eventually leads to death among pneumoconiosis patients and others suffering from chronic respiratory diseases [9]. Under the ‘Enforcement Regulation of Classification and Examination of Pneumoconiosis Act’ in Korea, an investigation is carried out on the occasion of pneumoconiosis patient’s death to assess the causal relationship between the cause of death and pneumoconiosis. Accordingly, it is important to determine factors that contribute to death from pneumonia among these patients. To date, there have been several studies concerning various factors associated with pneumonia-mortality among patients with underlying pulmonary conditions such as chronic obstructive pulmonary disease (COPD) or interstitial lung disease [10–13]. However, for patients with pneumoconiosis, little is known about pneumonia-specific mortality since prior studies generally focused on epidemiologic outcomes just as the risk of all-cause mortality [3–5, 7, 8]. Owing to insufficient knowledge, when conducting an advisory investigation regarding causal relationship between mortality from pneumonia and pneumoconiosis, references have been commonly made to studies based on other chronic respiratory diseases mainly with COPD.

Considerable number of patients with pneumoconiosis die from pneumonia in Korea, yet, hardly any study was carried out to assess the risk factors associated with their deaths. Therefore, the aim of this study was to analyze factors associated with increasing the risk of mortality from pneumonia among patients with pneumoconiosis.

## Methods

### Data source and study population

The present study was performed by reviewing medical administration records of patients with pneumoconiosis who were hospitalized with pneumonia between August 1, 2011 and September 10, 2014, at the Department of Occupational and Environmental Medicine of Seoul St. Mary’s Hospital. Whole participants have been compensated for pneumoconiosis and registered at Korea Worker’s Compensation and Welfare Service by recorded working history and confirmed radiologic exam. Inclusion criteria was hospitalization of these patients due to pneumonia (*n* = 103). Exclusion Criteria were lung cancer (*n* = 7) or other malignancy and lack of data in medical records (*n* = 12). Patients who underwent cancer surgery was not excluded if there was no evidence of recurred or ongoing malignancy after the operation. Female patients (*n* = 2) were excluded due to small number of subjects to analyze. The number of final eligible subjects was 82.

### Case definition

With regard to inclusion criteria, patients hospitalized for pneumonia during the referred period were considered. Diagnosis as pneumonia was done by medical examination, blood tests and radiologic exam. In the medical record, ICD (International Classification of Diseases)-10-code J12x, J13x, J15x, J17x, J18x were counted as pneumonia. The participants were divided into two groups by clinical outcome of pneumonia as follows; the patients who survived pneumonia and the others patients who died from pneumonia.

### Radiologic findings of subjects

Radiographic progression of pneumoconiosis was assessed according to International Labor Organization (ILO) classification [14]. The extent of small opacities was categorized into profusion score (1, 2 or 3) based on a three-point scale. Large opacities, exceeding width of 10 mm, were classified into either A, B or C. The classification of large opacities A cover those of which longest dimension up to 50 mm or several large opacities with the sum of their longest dimension. Large opacities exceeding width of 50 mm but not exceeding the equivalent area of the right upper lung (RUL) zone was classified as B. If the sum of size of large opacities exceeded equivalent area of RUL zone, it was classified as C.

Whether the patient had fungal ball infection or interstitial lung fibrosis was also determined. Fungal ball infection was confirmed with sputum culture and computed tomography (CT) of the chest. Existence of concomitant interstitial lung fibrosis with pneumoconiosis was acknowledged only when the chest CT showed either a finding of interstitial lung disease (ILD), interstitial pulmonary fibrosis (IPF), non-specific interstitial fibrosis or fibrotic type pneumoconiosis.

### Demographic and clinical characteristics

Each patients’ general and clinical characteristics including age at the diagnosis of pneumonia, smoking history, results of spirometry and episodes of recent pneumonia were reviewed and assessed. Recent pneumonia was defined as history of recent hospitalization with pneumonia in 90 days from the last day of hospitalization with pneumonia. In addition, type of pneumoconiosis was taken into account depending upon the type of suspected dust which subjects had been exposed to. All of subjects were classified as either silicosis or coal worker’s pneumoconiosis (CWP). There was no patient who was suspicious of asbestosis or other type of pneumoconiosis based on occupational history.

The results values of the latest spirometry before the case of pneumonia was used for the analysis. In spirometry, the percentage values of predicted forced expiratory volume in one second (FEV_1_ % = measured FEV_1_/reference FEV_1_), forced vital capacity (FVC % = measured FVC/reference FVC) and FEV_1_-FVC ratio (FEV_1_/FVC) were checked. The reference values were calculated using *Choi Jung-Keun* equation [15]. Licensed and trained technicians performed the test using a dry rolling seal spirometer (model 2130; SensorMedics, Yorba Linda, CA, USA). The quality-control program was fulfilled under calibration and complying with the American Thoracic Society/European Respiratory Society criteria of acceptability and repeatability of the spirometry [16].

### Statistical analysis

The two groups were compared in terms of demographic, radiologic and clinicopathological variables. Continuous variables including age, smoking history as pack-years (number of packs smoked per days multiplied by the number of years as a smoker) and the measured values of spirometry followed normal distributions and they were compared between the two groups using student *t*-test. Chi-square test was used to analyze the differences in history of recent pneumonia, extent of small opacities, grade of large opacities and presence of interstitial fibrosis or fungal ball infection between two groups. Fisher’s exact test was performed to evaluate differences for factors including FEV_1_ less than 70 % of predicted value, FVC less than 70 % of predicted value, type of pneumoconiosis and concomitancy of fungal ball. In multiple logistic regression analysis, age, smoking history, episode of recent pneumonia, concomitancy of interstitial fibrosis or fungal ball infection, extent of small opacities, grade of large opacities, type of pneumoconiosis and results of spirometry (FEV_1_ % or FVC %) were adjusted.

Statistical Analysis System (SAS) version 9.3 (SAS Institute, Cary, NC, USA) was used to analyze the data. The ethical issues of the study was approved by the Institutional Review Board Seoul St. Mary’s Hospital (ID: KC14RISI0831).

## Results

Table 1 describes general characteristics of the subjects and the differences between the two groups. Mean values are presented in bold type with standard deviation following ± mark. Categorical values are presented with number of the case followed by percentage in brackets. The total mean age of the patients was 71.0 ± 7.2 years. Sixty-two patients were ex-smokers and four patients were current-smoker. Comparing with the survival group, the deceased group showed statistically significant decrease in FEV_1_ % and FVC %. Those who experienced recent pneumonia in the preceding three months were more likely to expire from pneumonia. Also, there were more frequent presence of interstitial fibrosis or fungal ball infection in the deceased group.

**Table 1:** Demographic and clinical features of the study population

	Total	Clinical outcome		
		Survival	Decease	*p*-value
Age	**71.0 ± 7.2**	**71.4 ± 7.1**	**70.0 ± 7.4**	0.456
50–59		5 (8.2 %)	1 (4.8 %)	
60–69		14 (23.0 %)	10 (47.6 %)	
70–79		39 (63.9 %)	8 (38.1 %)	
Over 80		3 (4.9 %)	2 (9.5 %)	0.119
Smoking history				
Pack-years^a^	**18.0 ± 18.1**	**15.9 ± 16.9**	**24.2 ± 20.3**	0.068
Never smoker		14 (23.0 %)	2 (9.5 %)	
Previous smoker		44 (72.1 %)	18 (85.7 %)	
Current smoker		3 (4.9 %)	1 (4.8 %)	0.401
Pneumoconiosis characteristics^b^				
Profusion of small opacity				
1		15 (24.6 %)	2 (9.5 %)	
2		37 (60.7 %)	13 (62.0 %)	
3		9 (14.8 %)	6 (28.6 %)	0.188
Grade of large opacity				
No		17 (27.9 %)	3 (14.3 %)	
A		8 (13.1 %)	4 (19.0 %)	
B		22 (36.1 %)	10 (47.6 %)	
C		14 (23.0 %)	4 (19.0 %)	0.532
Pulmonary function profiles				
FEV_1_ % predicted	**50.9 ± 20.6**	**53.6 ± 22.1**	**43.1 ± 13.4**	0.012
70 ≤		13 (21.3 %)	1 (4.8 %)	
< 70		48 (78.7 %)	20 (95.2 %)	0.102
FVC % predicted	**63.4 ± 17.7**	**66.9 ± 18.1**	**53.4 ± 11.9**	<0.001
70 ≤		25 (40.9 %)	2 (9.5 %)	
< 70		36 (59.0 %)	19 (90.5 %)	0.008
FEV_1_/FVC ratio	**58.3 ± 18.4**	**57.8 ± 18.0**	**59.9 ± 19.9**	0.648
70 ≤		19 (31.1 %)	8 (38.1 %)	
< 70		42 (68.9 %)	13 (61.9 %)	0.559
Type of pneumoconiosis				
silicosis		14 (22.9 %)	3 (14.3 %)	
CWP^c^		47 (77.1 %)	18 (85.7 %)	0.839
Recent pneumonia^d^				
No		46 (75.4 %)	9 (42.9 %)	
Yes		15 (24.6 %)	12 (57.1 %)	0.006
Interstitial fibrosis^e^				
No		54 (88.5 %)	13 (61.9%)	
Yes		7 (11.5 %)	8 (38.1 %)	0.007
Fungal ball^f^				
No		59 (96.7 %)	18 (85.7 %)	
Yes		2 (3.3 %)	3 (14.3 %)	0.103
Hospitalized days	**14.8 ± 13.5**	**13.0 ± 13.4**	**19.9 ± 13.0**	0.044
Total	82 (100 %)	61 (74.4 %)	21 (25.6 %)	

Mean values are shown in bold type with standard deviation following ± mark. Categorical values are presented with number of the case followed by percentage in brackets

^a^Pack-years: Pack-years of never smokers were counted as 0

^b^Pneumoconiosis characteristics: Radiographic findings assessed in accordance with ILO classification

^c^CWP : Coal worker’s pneumoconiosis

^d^Recent pneumonia: History of recent hospitalization due to pneumonia within 90 days from the last day of hospitalization with pneumonia

^e^Interstitial fibrosis: Presence of interstitial fibrosis confirmed by findings of chest CT including ILD, IPF, non-specific interstitial fibrosis or fibrotic type pneumoconiosis

^f^Fungal ball: Concomitancy of fungal ball infection confirmed by sputum culture and chest CT

Table 2 summarize the results of logistic regression analysis. The crude ORs for recent pneumonia, interstitial fibrosis and FVC less than 70 % of predicted value were 4.09 (95 % CI 1.46-11.95), 4.75 (95 % CI 1.46-15.99) and 6.60 (95 % CI 1.70-43.75), respectively. In Model 1, adjusting for age, smoking history, recent pneumonia, interstitial fibrosis, fungal ball, type of pneumoconiosis, large opacity, profusion and FVC less than 70 % of predicted, ORs for recent pneumonia, interstitial fibrosis, fungal ball, CWP and FVC less than 70 % of predicted value were 6.33 (95 % CI 1.52-31.12), 6.50 (95 % CI 1.41-35.82), 73.44 (95 % CI 3.69- > 999.99), 7.59 (95 % CI 1.19-74.73) and 16.86 (95 % CI 1.19-74.73), respectively. In Model 2, age, smoking history, recent pneumonia, interstitial fibrosis, type of pneumoconiosis, fungal ball, large opacity, profusion and FEV_1_ less than 70 % of predicted were adjusted. Statistically significant risk elevation of pneumonia-mortality was detected in history of recent pneumonia (OR 6.47, 95 % CI 1.64-29.99), interstitial fibrosis (OR 8.91, 95 % CI 1.95-52.63), fungal ball (OR 13.75, 95 % CI 1.30-210.44) and CWP (OR 7.76, 95 % CI 1.21-79.15). FEV_1_ less than 70 % of predicted value also showed elevated OR of 10.95 (95 % CI 1.11-302.02).

**Table 2:** Factors associated with pneumonia-mortality among subjects

	Unadjusted model		Model 1		Model 2	
	OR	95 % CI	OR	95 % CI	OR	95 % CI
Age (per year)	0.97	0.91-1.04	0.98	0.89-1.08	0.99	0.91-1.09
Smoking (pack-years)	1.03	0.99-1.05	1.03	0.99-1.07	1.03	0.99-1.07
Recent pneumonia						
No	1.00		1.00		1.00	
Yes	**4.09**	**1.46-11.95**	**6.33**	**1.52-31.12**	**6.47**	**1.64-29.99**
Interstitial fibrosis						
No	1.00		1.00		1.00	
Yes	**4.75**	**1.46-15.99**	**6.50**	**1.41-35.82**	**8.91**	**1.95-52.63**
Fungal ball						
No	1.00		1.00		1.00	
Yes	4.92	0.76-39.58	**73.44**	**3.69- > 999.99**	**28.47**	**2.18-654.13**
Type of pneumoconiosis						
Silicosis	1.00		1.00		1.00	
CWP	1.79	0.51-8.41	**7.59**	**1.19-74.73**	**7.76**	**1.21-79.15**
Large opacity						
No	1.00		1.00		1.00	
A or B or C	2.32	0.67-10.79	3.07	0.46-30.23	1.99	0.33-15.20
Profusion						
1	1.00		1.00		1.00	
2	2.64	0.63-18.16	1.32	0.15-15.47	1.04	0.12-12.28
3	5.00	0.93-39.49	1.83	0.17-26.28	1.93	0.18-28.08
FEV_1_ predicted (%)						
≥ 70	1.00		NA		1.00	
< 70	5.42	0.98-101.65	NA		**10.95**	**1.11-302.02**
FVC predicted (%)						
≥ 70	1.00		1.00		NA	
< 70	**6.60**	**1.71-43.75**	**16.86**	**1.19-74.73**	NA	

Statistically significant values are shown in bold type

Model 1: Adjustment for age, smoking history, recent pneumonia, fungal ball, type of dust, large opacity, profusion and FVC less than 70 % of predicted

Model 2: Adjustment for age, smoking history, recent pneumonia, fungal ball, type of dust, large opacity, profusion and FEV1 less than 70 % of predicted

## Discussion

In the present study, the concomitancy of interstitial fibrosis or fungal ball, history of recent pneumonia within last 90 days, type of pneumoconiosis, FEV_1_ less than 70 % of predicted value and FVC less than 70 % of predicted value showed statistically significant positive associations with pneumonia-mortality.

History of recent pneumonia within last 90 days was shown to be significantly associated with pneumonia-mortality in the present study. It is accordant to previous researches that people who have medical record of recent admission for pneumonia tends to be re-hospitalized for pneumonia compared to the others who don’t [17, 18]. The preceding infection of respiratory tract has long been suspected as a predisposing factor for pneumonia [19]. The present study adds a concept that recent admission for pneumonia is not only associated with subsequent re-hospitalization for pneumonia but also with poor clinical outcome of pneumonia as well. Patients with pneumoconiosis commonly get pneumonia, and frequent occur of pneumonia may imply more likelihood of death from it. To confirm, further studies should be followed to determine factors associated with frequent occurrence of pneumonia.

The host defense system acts against respiratory pathogens to prevent occurrence of lung infection and defense agents such as macrophage, lymphocytes and mucociliary cells play a key role in the respiratory defense mechanism [17, 20, 21]. However, such respiratory defense system seems to be deteriorated among the patients with pneumoconiosis. According to previous studies, alveolar macrophages are directly damaged by silica or coal dust particles, followed by diverse inflammatory cytokines and free radicals derived directly from coal mine dust and indirectly from involved leukocytes [22, 23]. Subsequently, these reactions trigger fibrosis and impairment of lymphatic drainage of the lung. Therefore, it is hard to expect well-functioning defense mechanism from the lung of patients with pneumoconiosis. Aspergillus related lung disease is well known to be found in immunocompromised patients and tends to form mycelial balls in the lungs where the local defense mechanism is impaired with preexisting pulmonary disease [24–27]. In patients with complicated pneumoconiosis, central cavitation of progressive massive fibrosis often occurs due to ischemic necrosis or other conditions [28]. Development of fungal ball in this cavitary lesion can be source of hemoptysis or pulmonary infection when involved with invasive aspergillosis [26]. Therefore, the presence of fungal ball on Chest CT may indicate impaired respiratory defense mechanism of the patient with pneumoconiosis and lead to poor clinical course concerning pneumonia.

Despite showing statistically non-significant association in its simple regression analysis, type of pneumoconiosis (CWP versus silicosis) presented significantly increased odd ratio in both Model 1 and Model 2. There is few study concerning the difference in outcome of a pneumonia course between those diagnosed with CWP and silicosis. There would be several possible causes for those differences. In subgroup analysis, mean age of subjects with CWP (71.9 ± 7.3) was significantly higher than mean age of subjects with silicosis (67.7 ± 5.8) performing *t*-test (*p*-value = 0.03). This inequality may explain circumstances which led to higher mortality of subjects with CWP, though age was adjusted in Model 1 and Model 2. In addition, there is possibility of misclassification since occupational history was based on self-report. Still, there is little clue to clarify the correlation between higher pneumonia-mortality and CWP against silicosis. Accordingly, further study with proper design is required to investigate association between types of pneumoconiosis and pneumonia-mortality.

For the presence of interstitial fibrosis on the chest CT, we found an elevated risk for poor prognosis. This results corresponds to the GAP model recently introduced by Ley et al., which is used to predict mortality of idiopathic pulmonary fibrosis patients [29]. In the GAP model, multidimensional indices consist of gender(G), age(A) and 2 lung physiological factors(P) including FVC, which accounts for two points of total eight possible points [29]. The less percentage value of predicted FVC scores higher points, resulting in advanced stage and prediction of higher mortality [29]. Idiopathic pulmonary fibrosis generally shows ventilatory dysfunction with restrictive pattern. Interestingly, FVC less than 70 % of predicted value, which can be interpreted as a presence of restrictive ventilatory defect, was shown to be associated with increased mortality from pneumonia. It seems plausible to assume that pneumoconiosis patients with decreased baseline lung function and restrictive ventilatory impairment have disadvantages on the clinical course of pneumonia.

FEV_1_ less than 70 % of predicted value showed statistically significant result in Model 2. Decrement in FEV_1_ indicates lower exercise capacity [30] and higher comorbidities and mortality [31] in COPD patients. Loss of FEV_1_ may be also related with higher mortality from pneumonia among patients with pneumoconiosis. However, most of subjects in this study did not undergo post-bronchodilator test on their spirometry, making the value of FEV_1_ less reliable. Moreover, FEV_1_/FVC value could not be used to determining presence of obstructive ventilator impairment, or COPD. It is recommended to perform the test after using an adequate dose of a short-acting inhaled bronchodilator in order to minimize variability [31]. Due to retrospective study design, it was not possible to gain the result of spirometry after administration of a bronchodilator and further analysis or assay was not feasible with retained records of spirometry. To minimize systematic error and bias, utilizing post-bronchodilator results of spirometry is needed in further study.

Predicted values of FVC and FEV_1_ were derived using *Choi Jung-Keun* equation [15], however, there is a newer spirometric reference equation for Korean population [32]. As a matter of fact, the present ‘Korea worker’s compensation and welfare service’ is officially recommending *Choi Jung-Keun* equation for spirometric reference in worker’s specific examination. This was the main reason why the antecedent reference equation was applied despite acknowledging a newer reference equation. Additional analyses were performed to confirm if there was any consequence of applying the newer reference equation. After employing the newer reference equation, FEV_1_ less than 70 % of predicted value turned out to be statistically significant factor in unadjusted model (OR 6.52, 95 % CI 1.19-121.88). It remained statistically significant (OR 11.59, 95 % CI 1.17-315.09) after adjusting other variables in Model 2. FVC less than 70 % of predicted value remained statistically significant after applying the newer reference in both unadjusted model (OR 3.55, 95 % CI 1.28-10.62) and Model 2 (OR 12.79, 95 % CI 2.28-108.46). Notably, other variables such as history of recent pneumonia, concomitancy of fungal ball and presence of interstitial fibrosis remained statistically significant in both Model 1 and Model 2, regardless of choice of reference equation.

The major limitations of this study was small number of participants and single center retrospective design. However, medical records of every patient with pneumoconiosis who admitted with pneumonia was thoroughly collected and reviewed for the analysis. Additionally, other pneumonia-mortality predicting variables such as initial vital sign or blood test indices were not included in the analysis. Even so, all patients who visited outpatient clinic or emergency room for pneumonia were assessed using CURB criteria, to determine the severity of pneumonia and predict the mortality [33–35]. Patients with CURB score equal to two or greater were hospitalized while the others were treated as outpatients. Following this rule, severities of pneumonia among subjects in this study resulted in less varied. Third, all subjects were male, owing to small number of female patients. Since this study was conducted in a single center, these results may have been affected by selection bias. Therefore, for confirmation on these findings and clearer assay, further multi-centered study with prospective design and consideration of other mortality-predicting factors are needed.

This is the first exclusive study that analyzed association between clinicopathological factors and mortality from pneumonia among the patients with pneumoconiosis in Korea. The findings could help clinicians to cure pneumonia of patients with pneumoconiosis, by paying more attention to those who have such proposed factors associated with pneumonia-mortality. Furthermore, it could be implemented to investigating whether the cause of death is related to pneumoconiosis when deciding to claim compensation in accordance with Enforcement Regulation of Classification and Examination of Pneumoconiosis Act.

## Conclusion

The concomitancy of fungal ball or interstitial fibrosis, history of recent pneumonia within last 90 days, type of pneumoconiosis, FVC less than 70 % of predicted value, FEV_1_ less than 70 % of predicted value presented statistically significant association with mortality from pneumonia. Review of medical history and exams should be done to detect such factors when treating pneumonia with pneumoconiosis.

## References

[1] Ortmeyer CE, Baier EJ, Crawford GM (1973). Life expectancy of Pennsylvania coal miners compensated for disability as affected by pneumoconiosis and ventilatory impairment. Arch Environ Health.

[2] Ortmeyer CE, Costello J, Morgan WK, Swecker S, Peterson M (1974). The mortality of Appalachian coal miners, 1963 to 1971. Arch Environ Health.

[3] Cochrane AL (1973). Relation between radiographic categories of coalworkers’ pneumoconiosis and expectation of life. Br Med J.

[4] Yi Q, Zhang Z (1996). The survival analyses of 2738 patients with simple pneumoconiosis. Occup Environ Med.

[5] Waters WE, Cochrane AL, Moore F (1974). Mortality in punctiform type of coalworkers’ pneumoconiosis. Br J Ind Med.

[6] Atuhaire LK, Campbell MJ, Cochrane AL, Jones M, Moore F (1986). Specific causes of death in miners and ex-miners of the Rhondda Fach 1950–80. Br J Ind Med.

[7] Miller BG, Jacobsen M (1985). Dust exposure, pneumoconiosis, and mortality of coalminers. Br J Ind Med.

[8] Sadler RL, Roy TJ (1990). Smoking and mortality from coalworkers’ pneumoconiosis. Br J Ind Med.

[9] Meijers JM, Swaen GM, Slangen JJ (1997). Mortality of Dutch coal miners in relation to pneumoconiosis, chronic obstructive pulmonary disease, and lung function. Occup Environ Med.

[10] Fumeaux T, Rothmeier C, Jolliet P (2001). Outcome of mechanical ventilation for acute respiratory failure in patients with pulmonary fibrosis. Intensive Care Med.

[11] Gomez-Junyent J, Garcia-Vidal C, Viasus D, Millat-Martinez P, Simonetti A, Santos MS (2014). Clinical features, etiology and outcomes of community-acquired pneumonia in patients with chronic obstructive pulmonary disease. PLoS One.

[12] Stern JB, Mal H, Groussard O, Brugiere O, Marceau A, Jebrak G (2001). Prognosis of patients with advanced idiopathic pulmonary fibrosis requiring mechanical ventilation for acute respiratory failure. Chest.

[13] Loke YK, Kwok CS, Wong JM, Sankaran P, Myint PK (2013). Chronic obstructive pulmonary disease and mortality from pneumonia: meta-analysis. Int J Clin Pract.

[14] International Labour Office (2011). Guidelines for the use of the ILO International Classification of Radiographs of Pneumoconioses.

[15] Choi JK, Paek D, Lee JO (2005). Normal Predictive Values of Spirometry in Korean Population. Tuberc Respir Dis.

[16] Miller MR, Hankinson J, Brusasco V, Burgos F, Casaburi R, Coates A (2005). Standardisation of spirometry. Eur Respir J.

[17] Almirall J, Bolibar I, Balanzo X, Gonzalez CA (1999). Risk factors for community-acquired pneumonia in adults: a population-based case–control study. Eur Respir J.

[18] Hedlund JU, Ortqvist AB, Kalin M, Scalia-Tomba G, Giesecke J (1992). Risk of pneumonia in patients previously treated in hospital for pneumonia. Lancet.

[19] Rose RM, Pinkston P, O′Donnell C, Jensen WA (1987). Viral infection of the lower respiratory tract. Clin Chest Med.

[20] Reynolds HY (1987). Host defense impairments that may lead to respiratory infections. Clin Chest Med.

[21] Segal LN, Rom WN, Weiden MD (2014). Lung microbiome for clinicians. New discoveries about bugs in healthy and diseased lungs. Ann Am Thorac Soc.

[22] Fujimura N (2000). Pathology and pathophysiology of pneumoconiosis. Curr Opin Pulm Med.

[23] Dalal NS, Suryan MM, Vallyathan V, Green FH, Jafari B, Wheeler R (1989). Detection of reactive free radicals in fresh coal mine dust and their implication for pulmonary injury. Ann Occup Hyg.

[24] Barnes PD, Marr KA (2006). Aspergillosis: spectrum of disease, diagnosis, and treatment. Infect Dis Clin North Am.

[25] Denning DW (1998). Invasive aspergillosis. Clin Infect Dis.

[26] Segal BH (2009). Aspergillosis. N Engl J Med.

[27] Nomoto Y, Kuwano K, Hagimoto N, Kunitake R, Tsuda M, Hara N (1997). Aspergillus fumigatus Asp fI DNA is prevalent in sputum from patients with coal workers’ pneumoconiosis. Respiration.

[28] Chong S, Lee KS, Chung MJ, Han J, Kwon OJ, Kim TS (2006). Pneumoconiosis: comparison of imaging and pathologic findings. Radiographics.

[29] Ley B, Ryerson CJ, Vittinghoff E, Ryu JH, Tomassetti S, Lee JS (2012). A multidimensional index and staging system for idiopathic pulmonary fibrosis. Ann Intern Med.

[30] Wise RA (2006). The value of forced expiratory volume in 1 second decline in the assessment of chronic obstructive pulmonary disease progression. Am J Med.

[31] From the Global Strategy for the Diagnosis, Management and Prevention of COPD, Global Initiative for Chronic Obstructive Lung Disease (GOLD) 2016. Available from: http://www.goldcopd.org/. Accessed 3 Apr 2016.

[32] Eom SY, Kim H (2013). Reference values for the pulmonary function of Korean adults using the data of Korea National Health and Nutrition Examination Survey IV (2007–2009). J Korean Med Sci.

[33] Lim WS, van der Eerden MM, Laing R, Boersma WG, Karalus N, Town GI (2003). Defining community acquired pneumonia severity on presentation to hospital: an international derivation and validation study. Thorax.

[34] Howell MD, Donnino MW, Talmor D, Clardy P, Ngo L, Shapiro NI (2007). Performance of severity of illness scoring systems in emergency department patients with infection. Acad Emerg Med.

[35] Lim WS, Macfarlane JT, Boswell TC, Harrison TG, Rose D, Leinonen M (2001). Study of community acquired pneumonia aetiology (SCAPA) in adults admitted to hospital: implications for management guidelines. Thorax.

